# Surface Plasmon Enhanced Sensitive Detection for Possible Signature of Majorana Fermions via a Hybrid Semiconductor Quantum Dot-Metal Nanoparticle System

**DOI:** 10.1038/srep13518

**Published:** 2015-08-27

**Authors:** Hua-Jun Chen, Ka-Di Zhu

**Affiliations:** 1 Key Laboratory of Artificial Structures and Quantum Control (Ministry of Education), Department of Physics and Astronomy, Shanghai Jiao Tong University, Shanghai 200240, China; 2 Collaborative Innovation Center of Advanced Microstructures, Nanjing University, Nanjing 210093, China; 3 Department of Physics, Anhui University Of Science and Technology, Huainan Ahhui 232001, China

## Abstract

In the present work, we theoretically propose an optical scheme to detect the possible signature of Majorana fermions via the optical pump-probe spectroscopy, which is very different from the current tunneling measurement based on electrical methods. The scheme consists of a metal nanoparticle and a semiconductor quantum dot coupled to a hybrid semiconductor/superconductor heterostructures. The results show that the probe absorption spectrum of the quantum dot presents a distinct splitting due to the existence of Majorana fermions. Owing to surface plasmon enhanced effect, this splitting will be more obvious, which makes Majorana fermions more easy to be detectable. The technique proposed here open the door for new applications ranging from robust manipulation of Majorana fermions to quantum information processing based on Majorana fermions.

In recent years, nanostructures such as semiconductor quantum dots (SQDs) and metal nanoparticles (MNPs) have been made significant advances in modern nanoscience and nanotechnology for their applications in photonics and optoelectronics. SQD, as a simple stationary atom with tunability of optical properties^1^, paves the way for numerous potential applications^2^. Metal nanostructures can be excited to produce surface plasmon (SP) with strongly enhanced electromagnetic fields^3^,^4^,^5^. Currently, one hybrid system where SQDs coupled to MNPs has also attracted significant interest^3^,^6^,^7^. The SQD proximity to plasmonic nanostructures will induce significant alteration of the electromagnetic field felt by the SQD due to the interaction between the excitons in the SQD and the surface plasmons of the MNPs^8^,^9^,^10^. Owing to the exciton-plasmon interaction, several interesting phenomena, such as energy transfer^11^, local field enhancement^12^, intrinsic optical bistability^13^, and manipulation of population inversion^14^, have been explored in the hybrid complex SQD-MNP systems. In addition, the hybrid system provides an intuitive picture for highly sensitive detections and eventually leads to innovative new devices. Several applications including DNA sensors^15^, laser systems without cavities^16^, and manipulation of heat generation in MNPs^17^ have been investigated based on these complex systems. In the hybrid SQD-MNP system, the SPs have excellent ability to significantly improve the coherent optical responses^18^,^19^ and increase different kinds of physical processes^20^,^21^. Surface plasmon provides the enhanced nonlinear optical effects^22^ and the enhanced Raman scattering^23^.

On the other hand, Majorana fermions (MFs) have been received a great deal of attention recently^24^,^25^,^26^. A MF is a fermion that is its own antiparticle. This exotic particle obeys non-Abelian statistics and can be manipulated by braid operators to realize topological quantum computation^24^,^25^,^26^. Due to this promising application, the search and detection for MFs is currently under intensive studies. In the past few years, various systems that might host MFs in condensed matter systems have been proposed^27^,^28^,^29^,^30^,^31^,^32^,^33^ and many experimental attempts have also been devoted to identify them^34^,^35^,^36^,^37^,^38^,^39^,^40^. The zero-bias conductance peak^41^,^42^,^43^,^44^,^45^ as a distinct property of Majorana modes is often considered as a signature of MFs. These zero-bias conductance peaks have already been observed in several most recent experiments^36^,^37^,^38^,^39^,^40^. Whereas these experimental results can not serve as definitive evidences to prove the existence of MFs in condensed matter systems because the zero-bias conductance peaks can also appear in terms of the other mechanisms^46^,^47^,^48^,^49^, such as the zero-bias anomaly due to Kondo resonance^50^,^51^ and the disorder or band bending in the semiconductor nanowire (SNW)^52^. Most of the schemes to detect MFs are focusing on the electronic transport properties at present. Identifying MFs only through tunnel spectroscopy is somewhat problematic. Therefore, to obtain definitive signatures of MFs, alternative setups or proposals for detecting MFs are necessary.

For probing MFs, the nanometer QDs as intermediates are used to probe Majorana signature via tunneling experiments^53^,^54^,^55^,^56^,^57^,^58^. It is noticed that most of the schemes to detect MFs have been focused on the electronic transport properties, which can be significantly altered by MFs and will exhibit the signatures such as the zero-bias peak in the conductance^36^,^37^,^38^,^39^,^40^. However, other effective methods such as optical schemes for detecting MFs have received less attention until now. In the present work, based on the recent experiment by Mourik *et al.*^37^, in which a SNW with strong spin-orbit coupling placed in proximity with a superconductor (SC) under a proper external magnetic field (see Fig. 1), we propose an optical scheme to detect the possible signature of MFs. In our optical scheme, a hybrid SQD-MNP system with optical pump-probe scheme^59^,^60^ is introduced to detect MFs in the SNW/SC junction.

Compared with electrical detection schemes where the QDs are coupled to MFs via the tunneling^53^,^54^,^55^,^56^,^57^,^58^, in our optical scheme, there is no direct contact between the hybrid SQD-MNP system and the hybrid SNW/SC junction. The interaction between MFs in the SNW/SC junction and the SQD in the hybrid SQD-MNP system is mainly due to the dipole-dipole interaction. Since the distance between the hybrid SQD-MNP system and the hybrid SNW/SC junction can be adjusted by several tens of nanometers, therefore the tunneling between the SQD and MFs can be neglected safely. Once MFs appear in the hybrid SNW/SC devices and couple to the QD, the Majorana signature will be observed in the probe absorption spectrum. The change in the probe absorption spectrum as a possible signature for MFs is another potential evidence in the hybrid SNW/SC junction. This optical scheme will provide another method for the detection of MFs, which is very different from the zero-bias peak in the tunneling experiments^36^,^37^,^38^,^39^,^40^. Further, the surface plasmon enhanced effect will significantly enhance the probe absorption spectrum and eventually make MFs more sensitive to be detectable.

## Results

For illustration of the numerical results, we choose the realistic hybrid InAs SQD-Au MNP complex embedded in dielectric medium with constant permittivity *ε*_0_ = 1.8 and *S*_*α*_ = 2^61^ and the hybrid SNW/SC heterostructure^37^. For InAs SQD, we use the realistic experimental parameters^62^: *κ*_*SQD*_ ≈ 1.0 GHz, Γ_1_ = 2*κ*_*SQD*_, Γ_2_ = *κ*_*SQD*_, *μ* = *er*_0_ with *r*_0_ = 1 nm. The radius of Au MNP is *a*_0_ = 5 nm and its size-dependent dielectric function^63^ is 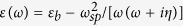 with 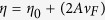, where *ε*_*b*_ = 9.5, *ħω*_*sp*_ = 9 eV, *ħη*_0_ = 0.07 eV, *A* = 0.25, and *v*_*F*_ = 1.4 nm/fs. Owing to dissipative losses in the metal, the SP mode displays a very fast relaxation time^6^. The SP mode relaxation rate used in the Au MNP is *κ*_*sp*_ ≈ 10.0 THz, and the coupling strength between the exciton and SP field is *g* ≈ 0.1 THz. As for MFs, there are no experimental values for the lifetime of the MFs and the coupling strength between the exciton in SQD and MFs in the recent literature. However, according to a few experimental reports^34^,^36^,^37^,^38^, it is reasonable to assume that the lifetime of the MFs is *κ*_*M*_ = 0.1 MHz. Since the coupling strength between the SQD and nearby MFs is dependent on their distance, we also expect the coupling strength *β* = 0.5 GHz via adjusting the distance between the hybrid SQD-MNP system and the hybrid SNW/SC heterostructure.

Firstly, we consider the case that there is no coupling between SQD and MNP (*g* = 0), i.e. only a single SQD couples to the nearby MF. Compared with the previous schemes to detect MFs via electrical measurements, the distinct difference of our scheme is focused on the optical detection. We first radiate a strong pump field and a weak probe field on the hybrid SQD-MNP system. Once the SQD is coupled to the nearby MF, the signature of MFs can be detected via the probe absorption spectrum. Figure 2(a) shows the absorption spectrum of the probe laser (i.e., the imaginary part of the dimensionless susceptibility Im*χ*^(1)^) as a function of the probe detuning Δ_*pr*_ (Δ_*pr*_ = *ω*_*pr*_ − *ω*_*ex*_) with (*β* = 0.2 GHz) and without (*β* = 0) the SQD-MF coupling. The black curve shows the result when there is no MFs in the nanowire. As the MFs appear in the ends of the nanowire, the probe absorption spectrum will present an asymmetric splitting at *∈*_*M*_ ≠ 0 (see the green curve). The physical origin of this result is that the SQD coupled to the nearby MF will induce the upper level of the state 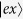 to split into 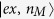 and 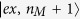 (*n*_*M*_ denotes the number states of the MFs). The left peak signifies the transition from 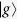 to 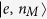 while the right peak is due to the transition of 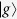 to 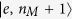 (as shown in the low two insets of Fig. 2(a)). To determine this signature is the true MFs that appear in the nanowire rather than the normal electrons that couple with the SQD, we give the numerical results of the normal electrons in the nanowire that couple with the SQD as shown in the upper right inset of Fig. 2(a). In order to compare with the MF, the parameters are chosen the same as MFs’ parameters. We find that there is no signal in the probe absorption spectrum (see the pink solid line in the inset of Fig. 2(a)) which means that the splitting of the probe absorption spectrum is the true signature of MFs. In addition, we further consider the condition of uncoupled MFs which means the two MFs are not coupled to each other, i.e., *∈*_*M*_ = 0, as shown in Fig. 2(b). In this situation, the coupled SQD-MF Hamiltonian will reduce to 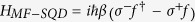. We can see that the probe absorption spectrum shows a symmetric splitting as the SQD-MF coupling strength *β* = 0.2 GHz and *∈*_*M*_ = 0 (see the blue curve of Fig. 2(b)). Therefore, our result reveals that the splitting in the probe absorption is a real signature of MF. The optical detection scheme can work at both the coupled Majorana edge states and the uncoupled Majorana edge states.

On the other hand, with increasing the SQD-MF coupling strength *β*, the distance of the splitting becomes larger and larger, and the stronger coupling strength induces the wider and deeper dip, which obviously reveals the SQD-MF coupling. In Fig. 3, the dip in the probe absorption spectrum goes to zero at Δ_*pr*_ ≈ 0, which means the input probe field is transmitted to the coupled system without absorption. Such a phenomenon is attributed to the destructive quantum interference effect between the Majorana modes and the beat of the two optical fields via the SQD. If the beat frequency of two lasers *δ* = *ω*_*pr*_ − *ω*_*pu*_ is close to the resonance frequency of MFs, the Majorana mode starts to oscillate coherently, which results in Stokes-like (Δ_*S*_ = *ω*_*pu*_ − *ω*_*M*_) and anti-Stokes-like (Δ_*AS*_ = *ω*_*pu*_ + *ω*_*M*_) scattering of light from the SQD. The Stokes-like scattering is strongly suppressed because it is highly off-resonant with the exciton frequency. However, the anti-Stokes-like field can interfere with the near-resonant probe beam and thus modify the signal beam spectrum. Here the Majorana modes play a vital role in this coupled system, and we can refer the above phenomenon as Majorana modes induced transparency, which is analogous with electromagnetically induced transparency (EIT) in atomic systems^64^.

Figure 3(a) shows the probe absorption spectrum as a function of detuning Δ_*pr*_ with three SQD-MF coupling constants at *∈*_*M*_ ≠ 0, and Fig. 3(b) gives the probe absorption spectrum under *∈*_*M*_ = 0. The figure shows that the distance of the two peaks in the probe absorption spectrum as a function of the SQD-MF coupling strength *β* follows a nearly linear relationship. This may provide a direct method to measure the SQD-MF coupling strength in this coupled system. The inset of Fig. 3 indicates the peak-splitting width as a function of the SQD-MF coupling strength *β* under the condition of the coupled MFs (*∈*_*M*_ ≠ 0) and the uncoupled MFs (*∈*_*M*_ = 0). It is obvious that the first two red dots (the uncoupled MFs) deviate significantly from the black line (the coupled MFs). However, the deviation becomes slighter with increasing in the coupling strength and finally the peak-splitting width is dependent linearly on the coupling strength. Therefore, it is essential to enhance the coupling strength for a clear peak-splitting via adjusting the distance between the SQD and the nearby MFs. In this case the coupling strength can also be obtained immediately by directly measuring the distance of two peaks in the probe absorption spectrum.

In the above discussions, we only consider that the relaxation rate of QD is *κ*_*SQD*_ ≈ 1.0 GHz (i.e. 5 times bigger than the coupling to the Majorana mode). In order to illustrate the effect of the relaxation rate *κ*_*SQD*_ on the probe absorption spectrum, Fig. 4 shows the spectrum for some relaxation rates of QD (*κ*_*SQD*_ = 0.1 GHz, 0.2 GHz, 0.5 GHz and 1.0 GHz). From the figure, we can see that although the order of magnitudes of *κ*_*SQD*_ is the same as the coupling to the Majorana mode, we can still obtain a significant dip in the probe absorption spectrum which indicates an evident signature of MFs in the hybrid SNW/SC heterostructure system. With increasing the relaxation rate *κ*_*SQD*_, the dip of the probe absorption spectrum becomes more observable. Therefore, although there are some fluctuations in the QD, the QD can be still considered as an effective probe for detection MFs. Once the QD is coupled to the MFs, the Majorana signature can be detected by the probe absorption spectrum of the QD.

In order to demonstrate the function of the MNP that enhances the sensitivity for detecting MFs, we should consider the effect of the SP in the hybrid SQD-MNP system. Figure 5(a) presents the absorption spectrum of the probe field as a function of the probe detuning Δ_*pr*_ with three SQD-MF coupling strengths (*β* = 0, 0.5 GHz, 1.0 GHz) under the exciton-SP coupling strengths *g* = 0.15 THz. It is obvious that the two peaks in the probe absorption spectrum present a large splitting, and with increasing the SQD-MF coupling strength *β*, the splitting of the two peaks of the probe absorption spectrum become more significant. There are two reasons induced such a phenomenon. The first one is the vacuum Rabi splitting in a single SQD induced by a MNP, which has been demonstrated theoretically in a hybrid SQD-MNP system^61^. In such a condition, the SP field is very similar to the optical cavity enhanced effect in quantum optics^65^, which will enhance the probe spectrum and make the MFs more sensitive to be detected. The second one is the SQD-MF coupling. Compared with the condition that the SQD-MF coupling *β* = 0 (the black curve), with increasing the SQD-MF coupling strength *β* (the blue and green curves), the SQD-MF coupling indeed induces the larger splitting. Return back to the quantum Langevin equations, we can see that Eqs (7) and (8) are symmetric with respect to the MFs and the SQD. As a consequence, the enhanced effect between the SQD-MF coupling and the exciton-SP coupling in the probe absorption spectrum is mutual.

In Fig. 5(a), the SP mode relaxation rate used in the Au MNP is *κ*_*sp*_ ≈ 0.1 THz. However, in ref. [6], the SP displays very fast relaxation times about at 1/*κ*_*sp*_ ~ 10 − 100 fs. Therefore, we further consider the situation of *κ*_*sp*_ ≈ 10 THz as shown in Fig. 5(b). In this circumstances, although there is exciton-SP coupling (*g* = 0.1 THz), the vacuum Rabi splitting still disappear and shows a usual Lorentzian line shape in the probe absorption spectrum under the SQD-MF coupling *β* = 0 (the black curve). Taking the SQD-MF coupling (*β* = 0.5 GHz) into account, a dip appears in the probe absorption spectrum, which indicates that MFs indeed appear in the nanowire and couples to the SQD (the red curve).

In Fig. 6, we further display the relaxation rate of the SP mode *κ*_*sp*_ that influences the detection of MFs with the hybrid SQD-MNP systems. Under the parameters of the SQD-MF coupling strength *β* = 0.5 GHz and the exciton-SP coupling strength *g* = 0.1 THz, the probe absorption spectrum presents different shapes with changing *κ*_*sp*_. The phenomenon is attributed to the destructive quantum interference effect between the Majorana modes and the beat of the two optical fields via the SQD. The inset of Fig. 6 shows the detail of the probe absorption spectrum in the narrow region around probe detuning Δ_*pr*_ ≈ 0. In this case, the results indicate that the influence of the SP mode relaxation rate *κ*_*sp*_ for detecting MFs via the hybrid SQD-MNP system is weak.

## Discussion

In this work, we have proposed an optical method to detect the existence of MFs in a hybrid semiconductor nanowire/superconductor structure via a single semiconductor quantum dot coupled to a metal nanoparticle. A direct scheme to determine the SQD-MF coupling strength is also demonstrated. Due to surface plasmon enhanced effect, the probe absorption spectrum becomes more significant and then enhances the detectable sensitivity of MFs.

On the other hand, there are several queries about our optical pump-probe technology for detecting MFs should be clarified. The first one is that how to restrain and differentiate the Zeeman splitting of the QD. Actually, the Zeeman effect is not good for detecting MFs using QDs with a single level in the electrical schemes. Because it will mix with the Majorana signature, and it is difficult to differentiate whether the observed signal is the true Majorana signature or induced by the Zeeman effect. However, in our optical proposal we have not taken the Zeeman effect into account, because the influence of the external magnetic field on the exciton in the InAs QD is small under the condition where Majorana zero energy states become observable around B = 0.15 T^37^. In addition, in real experiments, we can also choose the QDs with small g-factor in our scheme to distinguish InSb semiconductor nanowire in the hybrid superconductor-semiconductor nanowire devices. In this case the Zeeman effect will be small in these QDs, therefore we can neglect it in our optical proposal.

The other one is about how to differentiate other phenomena such as Kondo effect from Majorana signature. To detect MFs, a zero-bias anomalie (ZBA) in the tunneling spectroscopy is usually introduced and interpreted as the Majorana signature^36^,^37^,^38^,^39^,^40^. However, a ZBA might also occur under similar conditions due to a Kondo resonance that manifests when the magnetic field has suppressed the superconducting gap enough to permit the screening of a localized spin^39^,^50^. The Kondo effect usually stems from the antiferromagnetic coupling of a localized electron spin and a Fermi sea of conduction electrons^50^. Below a characteristic temperature *T*_*K*_ (the Kondo temperature), a many-body spin-singlet state is formed, leading to the partial or complete screening of the local magnetic moment. This phenomenon, discovered in metals containing diluted magnetic impurities, is now routinely found in individual QDs with a spin-degenerate ground state. For detecting MFs in the hybrid superconductor-semiconductor nanowire devices, the Kondo effect is usually associated with strong coupling to two normal leads in electrical detection scheme, and a superconducting gap is expected to suppress the effect when the gap is larger than the Kondo temperature.

Therefore, it is necessary to seek more definitive signatures of MFs with alternative detection means. Under the circumstances, we present an optical scheme for probing MFs with a hybrid semiconductor quantum dot-metal nanoparticle system. In our scheme, QD is considered as a two-level system without considering its spin-singlet state. When the optical pump-probe technology is applied on the QD modeled as a two-level system without considering its spin-singlet state, the detection of Majorana signature will be carried out via the probe absorption spectrum.

Most recently, Nadj-Perge *et al.* have reported the disappearance of edge-localized zero-bias peaks when the underlying superconductivity is suppressed. This will provide another evidence to show that the Majorana fermions is associated with superconductivity and not with other phenomena such as the Kondo effect^66^. Therefore, in order to restrain the Kondo effect the hybrid superconductor-semiconductor nanowire devices can also be replaced by this new scheme of a chain of Fe atoms fabricated on top of a superconductor Pb substrate to detect MFs.

In summary, the scheme proposed here may provide a potential application in all-optical controlled quantum devices based on MFs. Finally we hope that our proposed scheme can be realized experimentally in the near future.

## Methods

**Figure 1:**
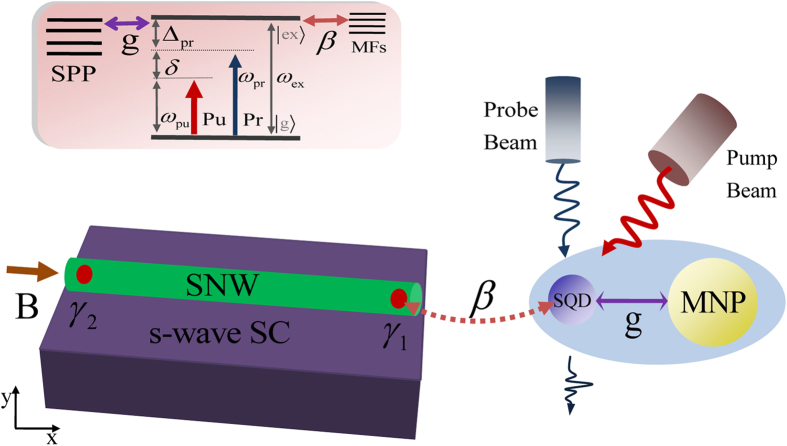
Schematic diagram of the proposed setup for optically detecting Majorana fermions (MFs). A semiconductor nanowire (SNW) in an external aligned parallel magnetic field (**B**) is placed on the surface of a bulk s-wave superconductor (SC). The two red spots at the ends of SNW represent a pair of MFs. The nearby MF is coupled to the exciton in the hybrid SQD-MNP system with optical pump-probe scheme. The inset is an energy-level diagram of the SQD coupled to MF and surface plasmons (SPs).

**Figure 2:**
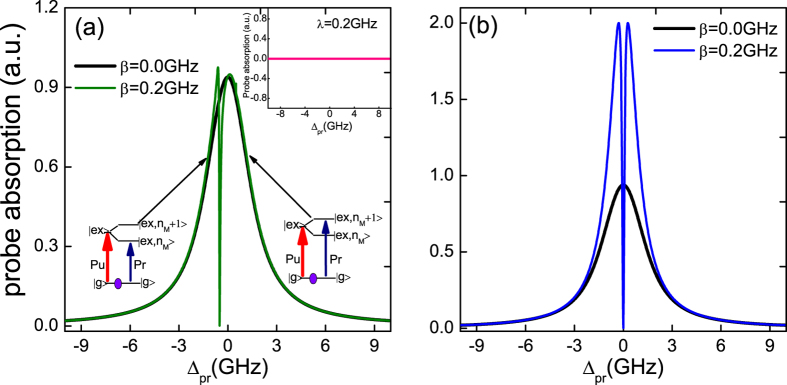
(a) The probe absorption spectrum as a function of detuning Δ_*pr*_ without and with SQD-MF coupling strengths *β* = 0 and *β* = 0.2 GHz at *∈*_*M*_ ≠ 0, respectively. The low two insets represents the energy level transitions of the left peak and right peak presented in the absorption spectrum. The top right corner inset is the normal electrons in the nanowire that couple with the SQD at the coupling strength 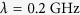. (**b**) The probe absorption spectrum without and with SQD-MF coupling strengths under *∈*_*M*_ = 0, respectively. The other parameters used are Γ_1_ = 2.0 GHz, Γ_2_ = 1.0 GHz, *κ*_*M*_ = 0.1 MHz, *κ*_*sp*_ = 10 THz, *g* = 0, 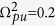 (GHz)^2^, and Δ_*pu*_ = Δ_*sp*_ = 0.

**Figure 3:**
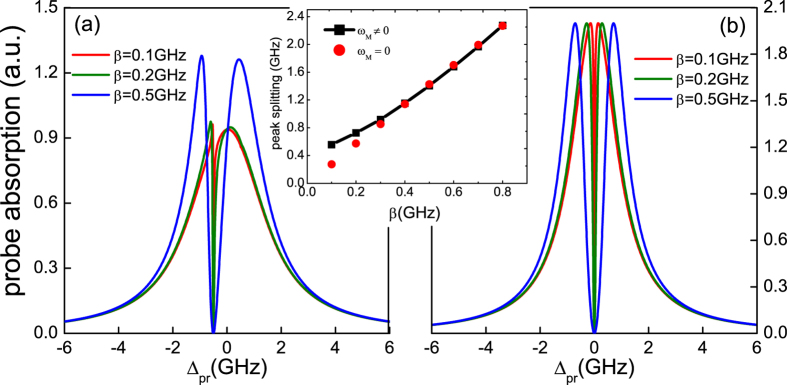
(a,b) show the probe absorption spectrum as a function of detuning Δ_*pr*_ with several different SQD-MF coupling strengths (*β* = 0.1 GHz, *β* = 0.2 GHz, *β* = 0.5 GHz) at *∈*_*M*_ ≠ 0 and *∈*_*M*_ = 0, respectively. The inset shows the linear relationship between the distance of peak splitting and the coupling strength of SQD-MF. The other parameters used are the same as in Fig. 2.

**Figure 4:**
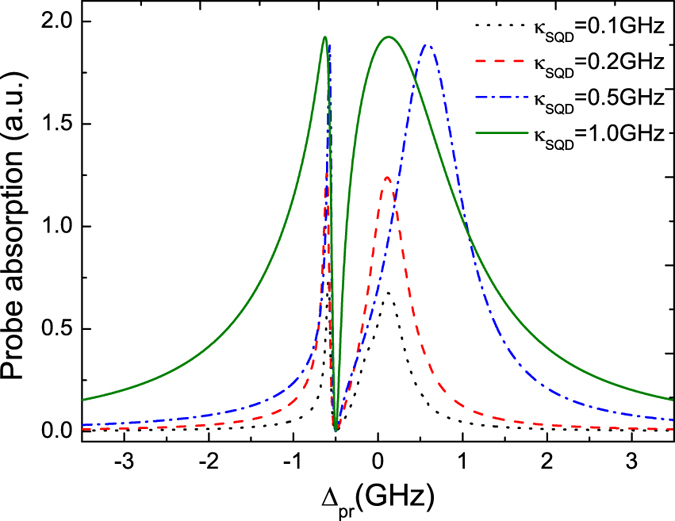
The probe absorption spectrum as a function of detuning Δ_*pr*_ for several *κ*_*SQD*_ with *β* = 0.2 GHz under *∈*_*M*_ ≠ 0. The other parameters used are the same as in Fig. 2.

**Figure 5:**
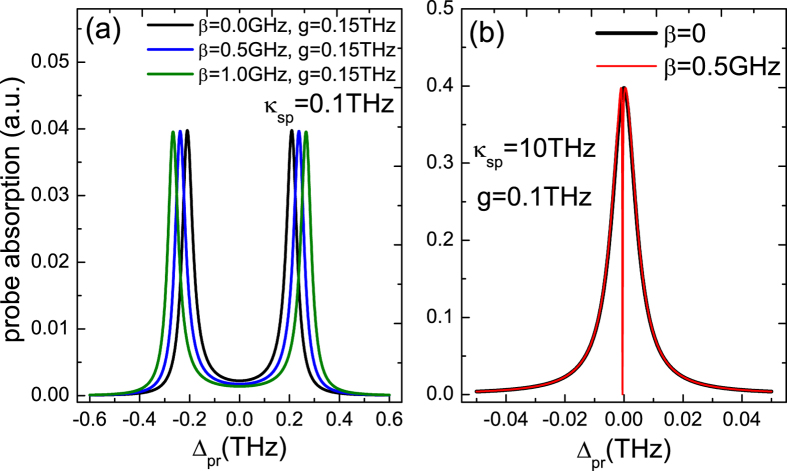
The probe absorption spectrum as a function of detuning Δ_*pr*_. (**a**) Three SQD-MF coupling strengths *β* = 0, 0.5 GHz, and 1.0 GHz at the exciton-SP coupling strengths *g* = 0.15 THz with *κ*_*sp*_ = 0.1 THz and *∈*_*M*_ ≠ 0. (**b**) The SQD-MF coupling *β* = 0 and 0.5 GHz at *g* = 0.1 THz, *κ*_*sp*_ = 10 THz, and *∈*_*M*_ ≠ 0. The other parameters used are the same as in Fig. 2.

**Figure 6:**
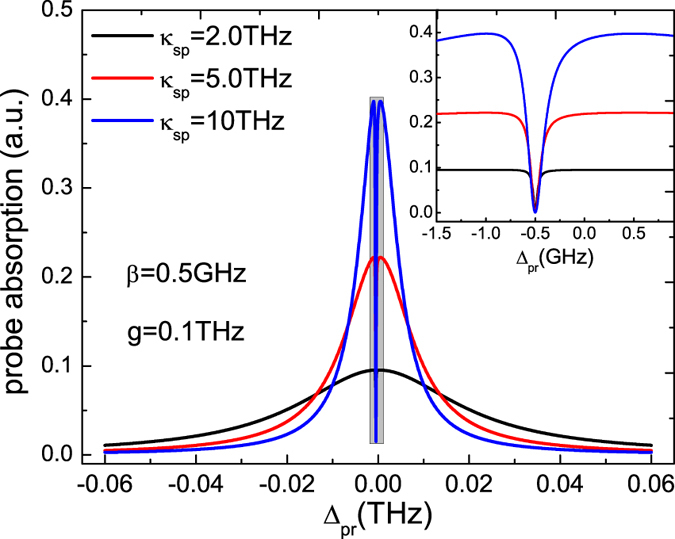
The probe absorption spectrum as a function of detuning Δ_*pr*_ with three *κ*_*sp*_ at *β* = 0.5 GHz, *g* = 0.1 THz, and *∈*_*M*_ ≠ 0. The other parameters used are the same as in Fig. 2.

Figure 1 shows the schematic setup that will be studied in this work. An InSb semiconductor nanowire with spin-orbit coupling in an external aligned parallel magnetic field **B** is placed on the surface of a bulk s-wave superconductor. A MF pair is expected to locate at the ends of SNW. Here we employ a hybrid SQD-MNP system as an optical sensitive probe to detect the existence of MFs in the SNW/SC junction. The inset of Fig. 1 shows the energy levels of the SQD coupled to the SP and MFs.

In the hybrid SQD-MNP system, the exciton in SQD can be modeled as a two-level system consisting of the ground state 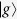 and the single exciton state 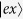 at low temperature^67^,^68^. The two-level exciton can be characterized by the operators *σ*^±^ and *σ*^*z*^ with the commutation relation 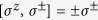 and 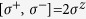. Then the Hamiltonian of the two-level exciton can be described as *H*_*ex*_ = *ħω*_*ex*_*σ*^*z*^ with exciton frequency *ω*_*ex*_. A MNP with a radius *a*_0_ couples to the SQD and the center-to-center distance between the SQD and MNP is *d*_0_. MNP can be excited to produce SP, which provides an external localized plasmon field^69^ and enhances the coherent optical properties of the SQD. There are two theoretical descriptions of the exciton-plasmon interaction in the hybrid SQD-MNP system at present, i.e., a semiclassical description^70^ and a full quantum description^7^,^71^. Since a quantum description for the exciton-plasmon interaction can reveal more novel quantum optical properties that may be applied in quantum processing devices, here we adopt the quantum description to the coupled SQD-MNP system. Quantizing the SP field in the MNP, the Hamiltonian can be written as *H*_*sp*_ = *ħω*_*sp*_*a*^+^*a*^7^,^61^,^72^, where *ω*_*sp*_ is the frequency of the SP mode, and *a* (*a*^+^) is its annihilation (creation) operator. The Hamiltonian term describing the interaction between the QD exciton and the quantized SP field is 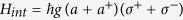. When we consider a rotating wave approximation, the Hamiltonian describing the exciton-SP field interaction reads 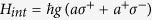 with the coupling strength *g*, which indicates the dipole-dipole interaction between the SQD and the MNP, and the term of non-conservation for energy 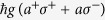 is generally neglected^7^,^61^. Therefore, the total Hamiltonian of the hybrid SQD-MNP system is given by
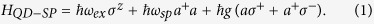


Although several experiments^34^,^35^,^36^,^37^,^38^,^39^,^40^ have been reported MFs in the hybrid SNW/SC heterostructure via electrical detection methods, there are still some debates whether the signatures are the definitive MFs. Hence the other schemes or proposals for probing MFs are indispensable. In what follows we will try to demonstrate the existence of MFs by using optical method, which can be considered as another supplement for detection of MFs. As each MF is its own antiparticle, an operator *γ* with *γ*^†^ = *γ* and *γ*^2^ = 1 to describe MFs is introduced. Supposed that the SQD couples to *γ*_1_, then the Hamiltonian is written by^53^,^54^,^55^,^56^,^57^,^58^
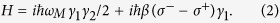


To detect MFs, it is helpful to switch the Majorana representation to the regular fermion one via the exact transformation *γ*_1_ = *f*^ †^ + *f* and *γ*_2_ = *i*(*f*^ †^ − *f*) , where *f* and *f*^ †^ are the fermion annihilation and creation operators obeying the anti-commutative relation {*f, f*^ †^} = 1. Accordingly, in the rotating wave approximation^7^, the above Hamiltonian can be rewritten as
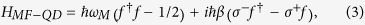
where the first term gives the energy of MF at frequency *ω*_*M*_, and *ħω*_*M*_ = *∈*_*M*_ ~ *e*^−l/*ξ*^ with the wire length (*l*) and the superconducting coherent length (*ξ*). This term is so small which can approach zero when the wire length is large enough. The second term describes the coupling between the nearby MF and the SQD with the coupling strength *β*, where the coupling strength is related to the distance between the hybrid SQD-MNP system and the hybrid SNW/SC heterostructure. It should be also noted that the term of non-conservation for energy, i.e. 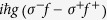, is generally neglected. We have made the numerical calculations (not shown in the following figures) and shown that the effect of this term is too small to be considered in our theoretical treatment.

Recently, the optical pump-probe technique has become a popular topic, which affords an effective way to investigate the light-matter interaction. The optical pump-probe technology includes a strong pump laser and a weak probe laser^73^. In the optical pump-probe technology, the strong pump laser is used to stimulate the system to generate coherent optical effect, while the weak laser plays the role of probe laser. Therefore, the linear and nonlinear optical effects can be observed via the probe absorption spectrum based on the optical pump-probe scheme. Xu *et al.* have obtained coherent optical spectroscopy of semiconductor quantum dot (SQDs) when driven simultaneously by two optical fields^59^,^60^. Their results open the way for the demonstration of numerous quantum level-based applications, such as QD lasers, optical modulators, and quantum logic devices. Most recently, this optical pump-probe scheme has also been realized experimentally in cavity optomechanical systems^74^,^75^,^76^, and several phenomena that include optomechanically induced transparency and the large change in light velocity, optically-tunable delay, and light storage have been demonstrated in different kinds of optomechanical systems based on the optical pump-probe technology. In terms of this scheme, we apply the pump-probe scheme to the SQD of the hybrid SQD-MNP system simultaneously. When the optical pump-probe technology is applied on the QD, the detection of Majorana signature will be carried out via the probe absorption spectrum.

The Hamiltonian of the exciton coupled to the two fields is given by^73^ HP–QD = – µΣi=pu,pr Ei 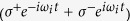, where *μ* is the dipole moment of the exciton, and *E*_*i*_ is the slowly varying envelope of the field. Therefore, we obtain the whole Hamiltonian of the hybrid system as H=HQD-SP  + 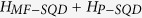. In a rotating frame at the pump field frequency *ω*_*pu*_, we obtain the total Hamiltonian of the system as
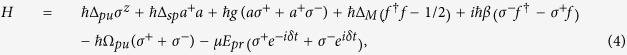
where 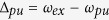 is the detuning of the exciton frequency and the pump frequency, 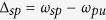 is the detuning of the SP and the pump frequency, 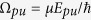 is the Rabi frequency of the pump field, and 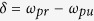 is the detuning of the probe field and the pump field. 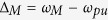 is the detuning of the MF frequency and the pump frequency. Actually, we have neglected the regular fermion like normal electrons in the nanowire that interact with the SQD in the above discussion. To describe the interaction between the normal electrons and the exciton in SQD, we use the tight binding Hamiltonian of the whole wire as^77^: 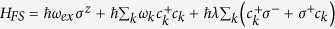, where *c*_*k*_ and 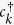 are the regular fermion annihilation and creation operators with energy *ω*_*k*_ and momentum *k* obeying the anti-commutative relation 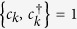, and *λ* is the coupling strength between the normal electrons and the exciton (here for simplicity we have neglected the *k*-dependence of *λ* as in ref. [78]).

According to the Heisenberg equation of motion and introducing the corresponding damping and noise terms, we derive the quantum Langevin equations^79^ as follows
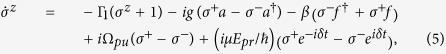

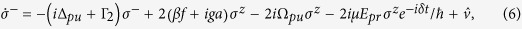

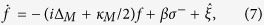

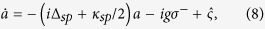
where Γ_1_ (Γ_2_) is the exciton spontaneous emission rate (dephasing rate), *κ*_*sp*_ is the SP mode relaxation rate and *κ*_*M*_ is the decay rate of the MF. 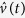 is the *δ*-correlated Langevin noise operator, which has zero mean 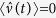 and obeys the correlation function 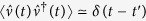. The MF is affected by a Brownian stochastic force with zero mean value 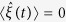, and 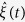 has the correlation function
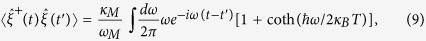
where *κ*_*B*_ and *T* are the Boltzmann constant and the temperature of the reservoir of the coupled system. The SP field has the same correlation relation 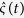 as MFs
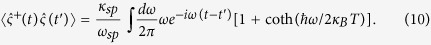


In Eq. (9) and Eq. (10), both the Majorana mode and SP mode will be affected by a thermal bath of Brownian and non-Markovian processes^79^. In the low temperature, the quantum effects of both the Majorana and SP mode are only observed in the case of 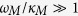 and 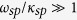. Due to the weak coupling to the thermal bath, the Brownian noise operator can be modeled as Markovian processes. In addition, both the SQD-MFs coupling and SQD-SP mode coupling in the hybrid system are stronger than the coupling to the reservoir that influences the two kinds’ coupling. In this case, owing to the second order approximation^79^, we can obtain the form of the reservoir that affects both the SP mode and Majorana mode as Eq. (9) and Eq. (10). And also, the quantum Zeno effect is too weak to be considered here.

To go beyond weak coupling, the Heisenberg operator can be rewritten as the sum of its steady-state mean value and a small fluctuation with zero mean value: *O* = *O*_0_ + *δO* (*O* = *σ*^*z*^, *σ*^−^, *f, a*). Inserting these operators into the Langevin equations (Eqs (5, 6, 7, 8)) and neglecting the nonlinear terms, we can obtain two equation sets about the steady-state mean value and the small fluctuation. The steady-state equation set consisting of *f*_0_, *a*_0_ and 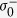 related to the population inversion 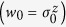 of the exciton is determined by
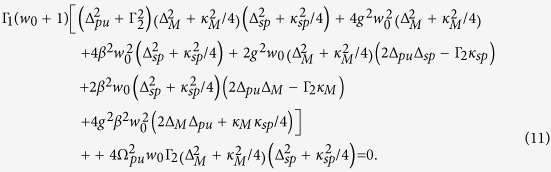


For the equation set of small fluctuation, we make the ansatz^73^ 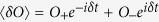. Solving the equation set and working to the lowest order in *E*_*pr*_ but to all orders in *E*_*pu*_, we can obtain the linear susceptibility as 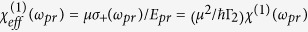, where 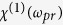 is given by
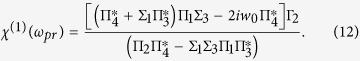
*f*_0_, 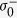 and *a*_0_ can be derived from the steady-state equations, and 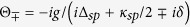, 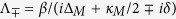, 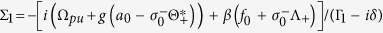, Σ2 = - 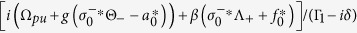, 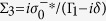, ∏1 = 2i(ga0      - Ωpu - ibf0), 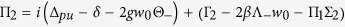, 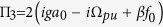, ∏4  = i(∆pu + δ 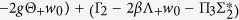 (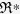 indicates the conjugate of 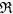). The imaginary and real parts of 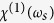 indicate absorption and dissipation, respectively. The quantum Langevin equations of the normal electrons coupled to the SQD have the same form as MFs, therefore, we neglect their derivations and only give the results in the following section.

## Additional Information

**How to cite this article**: Chen, H.-J. and Zhu, K.-D. Surface Plasmon Enhanced Sensitive Detection for Possible Signature of Majorana Fermions via a Hybrid Semiconductor Quantum Dot-Metal Nanoparticle System. *Sci. Rep.* **5**, 13518; doi: 10.1038/srep13518 (2015).
